# From mother to child: a survey-based cross-sectional study of oral health practices and knowledge among Saudi women during their pregnancy

**DOI:** 10.3389/fmed.2026.1911217

**Published:** 2026-08-11

**Authors:** Basim Almulhim

**Affiliations:** Department of Orthodontics and Pediatric Dentistry, College of Dentistry, Qassim University, Buraydah, Saudi Arabia

**Keywords:** dental practices, general health, knowledge, oral health, Saudi women

## Abstract

**Introduction:**

The overall and oral health status of a mother is reflected on her child. Before planning a pregnancy, completing any necessary dental treatment is critical. The current study aimed to investigate oral hygiene practices and knowledge among Saudi women by assessing oral health practices and knowledge, and the relationship between these factors and participants’ understanding of how general and oral health affect the health of their children.

**Methods:**

The study was a cross-sectional survey that utilized an online structured questionnaire. The questionnaire comprised questions related to oral hygiene practices and knowledge.

**Results:**

The total number of respondents included 448 Saudi women, with 45.8% aged between 21 and 30 years. Approximately 69.6% of respondents reported brushing their teeth, with 58.1% brushing at least once daily; 53.5% using a medium-bristle toothbrush, and 56.4% using other methods (miswak or dental floss). Approximately 64.3% understood the importance of vitamin supplements for the growth and development of their children, and 53.1% agreed that the oral health of a mother correlates with that of her child.

**Conclusion:**

Saudi women have adequate knowledge regarding maintaining their oral health, which was positively associated with better self-reported oral hygiene practices, and are aware that the overall health of a mother, including her oral health, influences the oral health of her child.

## 1 Introduction

The gestation period for the pregnant women is critical, with significant physiological and psychological changes in their endocrine system due to hormonal disturbances. Trying to keep and maintain proper oral hygiene during pregnancy is essential for both the mother and their child. However, it has been considered a public health problem (1, 2). Pregnant women may neglect oral hygiene practices during pregnancy owing to an increase in their physical and emotional stresses (2). Furthermore, other dental studies have revealed there is a strong, linked correlation between the mother’s intraoral health problems and adverse outcomes of pregnancy, such as eclampsia and preterm birth (3–8). Previous studies indicate that neglecting oral hygiene during pregnancy may lead to an increased risk of oral diseases related to dental or oral health, including tooth decay, gingival inflammation, or bone loss. Gingivitis, which manifests as gum bleeding, swelling, redness, or tenderness, is a more prevalent oral health issue faced by pregnant women and is usually observed during the fourth to sixth months of pregnancy (the second trimester), with greater involvement of the anterior than the posterior teeth (3–8). Researchers have documented a high occurrence of dental decay in the Saudi population (9, 10), with it being much more severe in children younger than 71 months (11, 12). Critical steps to minimize the risk of oral disease, including dental caries or other oral problems in young children in the early stage of life (early childhood caries), include increasing parental awareness through means such as caries prevention programs, oral health education, and modifying dietary patterns by changing social habits (13).

Neglecting maternal oral hygiene or leaving infections untreated is a significant risk factor for the overall health of the mother. Furthermore, an untreated infection poses more potential risks during pregnancy than any dental care (3–8). During pregnancy or breastfeeding, certain drugs may be needed to control pain or treat dental infection; while some are safe, others should be avoided. Paracetamol and amoxicillin, which are the most frequently used dental drugs in dental clinics, are among the safest drugs used during pregnancy or breastfeeding periods. However, certain drugs, such as aspirin, doxycycline, tetracycline, and benzodiazepines, possess potential risks for both mother and child and should be avoided. Furthermore, during pregnancy, women may require urgent dental care and local anesthesia, including common local anesthetics used for dental procedures, such as lidocaine with or without epinephrine (14, 15). Moreover, dental X-rays can be safely used during pregnancy provided proper techniques are employed, including the use of thyroid and abdominal shields and selecting the fastest image receptors, such as rare earth screen-film systems, digital imaging, and high-speed film, limiting re-exposure to protect the unborn child from radiation (16, 17). Typically, if the pregnant woman requires dental care, one should consider conducting elective dental restorative and periodontal procedures during the second trimester (16, 17).

Multiple studies conducted on pregnant women of various socioeconomic statuses, cultures, and races concerning practices related to oral hygiene and regular dental check-ups during pregnancy have indicated a lack of maternal understanding of the importance of dental visits and have revealed inadequate maternal general knowledge of the oral health issues faced during pregnancy (18, 19). Moreover, pregnant women should understand other pregnancy-related problems involving maternal health, such as pregnancy-induced anemia, hypertension, and premature delivery (20). Furthermore, nutrition plays a very critical role during pregnancy. The mother needs to receive the necessary nutrition during pregnancy; malnutrition can impact the overall health of a child, including oral health (21). The well-being of the unborn child generally requires pregnant women to consume a healthy diet (21). Additionally, during pregnancy, vitamin D levels are crucial, and if disturbed, they can affect the enamel integrity and normal development of the primary dentition of the fetus, thereby increasing the risk of teeth decay in the first years of child life, such as severe early childhood caries (22). B-complex vitamins, including folic acid, are also essential for child health, including cell synthesis, and they contribute to the development of the brain, spinal cord, and neural tube of newborns (17). Folic acid supplementation during pregnancy plays a critical role as a prevention factor in averting the risk of developing an oral cleft, including an isolated or combined cleft lip and palate (17). A recent study revealed that folic acid administration to mothers during the first 3 months of pregnancy (the first trimester) is crucial for preventing the development of cleft lip and palate (23). Two other dental studies focused on the oral health knowledge of mothers have been conducted in Saudi Arabia; one assessed the general pregnant women’s knowledge and their practices regarding oral health, whereas the other assessed the mothers’ understanding of their children’s oral health (24, 25). The current study aimed to investigate the oral healthcare practices and related knowledge of Saudi women during their pregnancy by assessing the association between maternal oral health practice and knowledge and the mother’s understanding of how her overall health, including oral health, may impact the oral health of her child.

## 2 Materials and methods

### 2.1 Study design, setting, and population

This study involved a cross-sectional descriptive survey conducted among Saudi women who were currently pregnant or had previously been pregnant. The study collected information regarding the oral healthcare practices and knowledge of these women with the goal of assessing the association between these two aspects and their understanding of how maternal overall health, including oral health, may impact the oral health of the child. The questionnaire was distributed between March 2022 and January 2023. The data of participants who returned an incomplete questionnaire were excluded from analysis.

### 2.2 Sample size

A power analysis was utilized to calculate the sample size using the Epi Info program, which was set to a significance level of 0.05, an effect size of 0.215, and a target power of 0.95. A total sample of 412 participants was required, accounting for a 10% expected nonresponse rate.

### 2.3 Data collection tools and procedure

A convenience sampling technique was used. Data were collected using an online survey distributed to mothers via a Google Form shared through the social media applications WhatsApp and Telegram. All participating women were invited to forward the survey link to other eligible participants (currently/previously pregnant women) within their networks. This method aimed to provide a useful and efficient way to access pregnant women who may be difficult to reach through traditional sampling methods. The questionnaire was developed originally in the English language, and then all the questionnaire items were translated into Arabic, the native language of the participants. To verify the accuracy and consistency of the translation, the questionnaire was reviewed by an independent bilingual reviewer to confirm and preserve the original meaning and content. The survey content was divided into several sections (three sections), including A, B, and C. Section A contained the demographic characteristics of the respondents. Section B assessed the oral health practices of the respondents. Section C evaluates the overall health knowledge of the respondents, including intraoral health, and assesses their understanding of how overall health may impact their child’s intraoral health. The content validity of the questionnaire was carefully reviewed and refined by the author in collaboration with an experienced pedodontist to ensure alignment with the study objectives. A reliability test was conducted and applied to assess and measure the internal consistency of the questionnaire questions by utilizing such psychometric testing as Cronbach’s alpha, providing scores for Section B (*α* = 0.710) and Section C (*α* = 0.804). Upon clicking the online survey link, a clear message displays detailed information regarding the researcher’s details, a brief summary of the research, including the study objectives, and the estimated time that may be required to finish the survey. All this information appeared at the top of the displayed page. Additionally, the inclusion criteria of the study (including only Saudi women who were currently pregnant or had previously been pregnant) were emphasized in bold font; moreover, we emphasized that participation was voluntary and that confidentiality would be maintained.

### 2.4 Statistical analysis

The overall data gathered were downloaded into a Microsoft Excel spreadsheet. The data were imported and analyzed utilizing the SPSS Statistics program version 25 (IBM Corp., Armonk, NY, United States). Categorical data variables are shown as frequencies and percentages, and quantitative data variables are shown as means and standard deviations. One-way analysis of variance (ANOVA), a *t*-test, and Scheffé’s multiple comparison test were utilized to assess the associations between demographic characteristics and oral hygiene practices and knowledge, with a significance threshold set at a level of *p* < 0.05.

### 2.5 Ethical considerations

The study was initially reviewed, and final approval was provided, by the Institutional Review Board committee at Majmaah University (approval no.: MUREC-May.13/COM-2020/29–3). The informed consent statement was displayed on the first page of the online survey upon clicking the link. Women confirmed their consent electronically by selecting “I agree to participate” before proceeding to the survey questions. Anonymity and confidentiality were preserved, as no personally identifiable information was collected.

## 3 Results

A total of 448 respondents provided complete responses to the online survey and were included and prepared for final analysis. The current study was organized into multiple sections. Section A involved the demographics of respondents from Saudi women. Section B aimed to investigate the practices of Saudi women regarding oral health. Section C aimed to evaluate the knowledge of Saudi women regarding overall health, including oral health, and their understanding of the impact of their health on the oral health of their children.

### 3.1 Respondent demographics

Table 1 summarizes the demographic variables of the respondents. The total number of respondents was 448, with the largest group (45.8%) falling between the ages of 21 and 30 years; close to half of the respondents (49.6%) reported having children; 55.1% of the Saudi women participants had at least a university degree (a bachelor’s degree); and 62.1% were unemployed.

**Table 1:** Demographic characteristics of the respondents.

Demographic variables	Level	*N*	%
Age	10–20 years	17	3.8
	21–30 years	205	45.8
	31–40 years	147	32.8
	41–50 years	70	15.6
	≥51 years	9	2.0
Pregnancy status/gestation period	Previously had children	222	49.6
	First trimester	83	18.5
	Second trimester	75	16.7
	Third trimester	68	15.2
Educational qualification	Elementary school	33	7.4
	Secondary school	119	26.6
	Bachelor’s degree	247	55.1
	Postgraduate degree	49	10.9
Occupation	Employed	170	37.9
	Housewife	278	62.1

N = number of respondents; % = percentage.

### 3.2 Oral health practices of respondents

Table 2 presents the Saudi women participants regarding their oral health practices. Approximately 69.6% of the Saudi women brushed their teeth, either at least once daily (58.1%) or by using a medium toothbrush (53.5%), whereas 30.4% did not brush their teeth, and 56.4% of the Saudi women used other methods for maintaining oral hygiene, such as the miswak or dental floss. Additionally, 44.6% of Saudi women rinsed their mouths infrequently, and 43.5% cleaned their tongue infrequently. Moreover, 63.6% of Saudi women visited a dentist when they experienced oral problems during pregnancy, whereas a small number of Saudi women visited the dentist regularly (12.9%).

**Table 2:** Oral-health practices of the respondents.

Factor	Level	*N*	%
1. Do you brush your teeth?	No	136	30.4
	Yes	312	69.6
2. If the answer is yes, how often?	Once daily	182	58.1
	Twice daily	115	36.7
	After every meal	16	5.1
3. What type of toothbrush do you use?	Soft	123	39.4
	Medium	167	53.5
	Hard	22	7.1
4. If no, then do you use any other oral hygiene method, such as miswak or dental floss?	No	171	43.6
	Yes	221	56.4
5. How often do you rinse your mouth?	Never	195	43.5
	Rarely	200	44.6
	Frequently	53	11.8
6. How often do you clean your tongue?	Never	182	40.6
	Rarely	195	43.5
	Frequently	71	15.8
7. How often do you visit a dentist?	Never	105	23.4
	Regularly	58	12.9
	Only when I experience oral problems during pregnancy	285	63.6

### 3.3 Association between the overall health knowledge of respondents, including oral health, and their understanding of the health impact on the oral health of the child

#### 3.3.1 Respondent general and oral health knowledge, regarding their health impact on the oral health of the child

Table 3 presents the knowledge of Saudi women participants regarding their overall health. Overall, 67.4% of the Saudi women were acutely aware of the importance of visiting a dentist before planning the pregnancy. The reported responses included completing all dental treatment procedures before pregnancy (46.1%), benefits for both the mother’s and child’s oral health (37.2%), and concerns that some medications may affect the teeth of their children (16.8%). Additionally, 57.4% of the respondents asserted the need for extra care toward oral hygiene during pregnancy, and 53.1% agreed that a significant correlation exists between the mother’s oral health and that of the child. Furthermore, most respondents (97.8%) were non-smokers, and 89.3% of the Saudi women believed that there is a strong correlation between oral cancer and smoking. Moreover, 64.5% of the respondents reported they had experienced oral health problems faced during pregnancy, with bleeding from the gingiva (37.2%) being the most common oral health problem, followed by tooth sensitivity (35.9%). Most respondents did not face a medical problem during pregnancy (74.6%); however, 25.4% of respondents reported facing certain medical issues, such as anemia, which was reported by the respondents as the most prevalent general health problem associated with pregnancy (45.9%). Of the respondents, 41.3% believed that the last periods of pregnancy (third trimester) were the safest time to receive dental care, whereas 40% preferred the middle periods of pregnancy (second trimester). Nearly 69.2% of respondents were aware that some drugs, such as aspirin, non-steroidal anti-inflammatory drugs, and tetracyclines, are not advisable during pregnancy; however, most of them (79.9%) believed that dental radiography and local anesthesia were unsafe during pregnancy. Moreover, 64.3% knew about the importance of supplementation of vitamins and minerals, such as B12, B6, K, D, and calcium, as well as folic acid, which may affect the normal development and growth of the child.

**Table 3:** Overview of the health knowledge of the respondents, including their understanding of how their health affects the oral health of their child.

Factor	Level	*N*	%
1. Is it important to visit a dentist before you plan for pregnancy?	No	146	32.6
	Yes	302	67.4
2. If the answer is yes, why?	To complete all dental treatment before pregnancy	140	46.1
	For mother and child oral health	113	37.2
	Some medications can affect the teeth of the child	51	16.8
3. Do you think that you should take more care of your oral hygiene during pregnancy?	No	191	42.6
	Yes	257	57.4
4. Do you think that there is a strong relationship between a mother’s oral health and a child’s oral health?	No	210	46.9
	Yes	238	53.1
5. Do you smoke?	No	438	97.8
	Yes	10	2.2
6. Do you think that there is a strong relationship between oral cancer and smoking?	No	48	10.7
	Yes	400	89.3
7. Did you experience oral health problems during pregnancy?	No	159	35.5
	Yes	289	64.5
8. If the answer is yes, what type of oral health problem did you experience?	Bleeding gums	108	37.2
	Swollen gums	31	10.7
	Gum redness	7	2.4
	Gum pain (tenderness)	27	9.3
	Teeth sensitivity	104	35.9
	Other	13	4.5
9. Did you experience any medical problems during pregnancy?	No	334	74.6
	Yes	114	25.4
10. If the answer is yes, what type of medical condition did you experience?	Diabetes	12	10.8
	Hypertension	21	18.9
	Anemia	51	45.9
	Others	27	24.3
11. What do you consider the safest trimester for dental treatment during pregnancy?	1st trimester	84	18.8
	2nd trimester	179	40.0
	3rd trimester	185	41.3
12. Are you aware that some medications, such as aspirin, NSAIDs, and tetracyclines, are not recommended for pregnant women?	No	138	30.8
	Yes	310	69.2
13. Do you know that dental radiography and local anesthesia are safe during pregnancy?	No	358	79.9
	Yes	90	20.1
14. Do you know how vitamins and supplements, such as B12, B6, K, Ca, D, and folic acid, during the first 3 months of pregnancy can affect a child’s growth and development, including conditions like ECC and cleft lip and palate?	No	160	35.7
	Yes	288	64.3

N = number of respondents; % = percentage; NSAIDs = non-steroidal anti-inflammatory drugs; Ca = calcium; ECC = early childhood caries.

#### 3.3.2 Relationship between respondent education levels and their oral hygiene practices and knowledge

Table 4 presents a summary of the results of a one-way ANOVA test to assess the different levels of education of the Saudi women in relation to their practices and knowledge toward oral hygiene. The average scores of practicing oral hygiene based on the different levels of Saudi women’s education ranged from 24.67 to 67.93. The percentage scores of oral hygiene practices were 24.67, 33.13, 55.81, and 67.93 for all levels of education: elementary, secondary schools, bachelor’s, and postgraduate degrees, respectively. The result of the one-way ANOVA test indicated a significant observation between the mean scores according to the education levels (*p*-value < 0.001). The Scheffé test indicated that the average scores of respondents with elementary and secondary school education were significantly lower than those of the respondents with bachelor’s and postgraduate degrees (*p*-value < 0.001), demonstrating a mean score of 67.93 for respondents with postgraduate degrees, which was significantly different from that of respondents with bachelor’s degrees (*p*-value = 0.021). Therefore, the oral hygiene practice scores increased significantly as education level increased.

**Table 4:** Associations between respondent education level and respondent oral hygiene practices and knowledge.

Variable	Education	*N*	Mean	Standard deviation	ANOVA *p*-value	95% Confidence interval		Multiple comparison test			
						Lower bound	Upper bound	Elementary school	Secondary school	Bachelor’s degree	Postgraduate degree
OHP % score	Elementary school	33	24.675	28.183	<0.001	14.682	34.669	1			
	Secondary school	119	33.133	26.232		28.371	37.895	0.389	1		
	Bachelor’s degree	247	55.813	24.488		52.744	58.882	<0.001	<0.001	1	
	Postgraduate degree	49	67.930	18.792		62.532	73.328	<0.001	<0.001	0.021	1
KOH % score	Elementary school	33	39.057	26.516	<0.001	29.655	48.459	1			
	Secondary school	119	48.086	26.082		43.351	52.821	0.277	1		
	Bachelor’s degree	247	64.957	22.381		62.152	67.762	<0.001	<0.001	1	
	Postgraduate degree	49	66.440	18.072		61.249	71.631	<0.001	<0.001	0.983	1

OHP = oral hygiene practice; KOH = knowledge of oral hygiene; ANOVA = analysis of variance.

The average scores of oral hygiene knowledge based on the level of education ranged from 39.057 to 66.440. The percentage scores of oral hygiene knowledge were 39.057, 48.086, 64.957, and 66.440 for all different levels of education: elementary, secondary schools, bachelor’s, and postgraduate degrees, respectively. The summary of the results of the one-way ANOVA test indicated a significant observation among the mean scores based on the different educational levels (*p*-value < 0.001). The results of Scheffé’s test indicated that the average scores of respondents with elementary and secondary school education were significantly lower than those of the respondents with bachelor’s and postgraduate degrees (*p*-value < 0.001). The respondents with postgraduate degrees had a mean score of 66.440, which was not significantly different from respondents with bachelor’s degrees (*p*-value = 0.983). Furthermore, the oral hygiene knowledge scores increased with an increasing level of education.

#### 3.3.3 The correlation between the practicing of oral hygiene and their knowledge among the respondents

Table 5 presents the results of the correlation between practicing oral hygiene and the knowledge of Saudi women. A positive significant correlation was noted between oral hygiene practices and knowledge, indicated by a correlation coefficient of 0.62 (62%) with a given *p*-value of <0.001, revealing that higher levels of knowledge toward oral hygiene were associated with better practices.

**Table 5:** Correlation between oral hygiene practice and knowledge of the respondents.

	OHP % score	KOH % score
OHP % score		
Pearson’s correlation	1	0.617**
Sig. (2-tailed)		<0.001
*N*	448	448
KOH % score		
Pearson’s correlation	0.617**	1
Sig. (2-tailed)	<0.001	
*N*	448	448

OHP = oral hygiene practice; KOH = knowledge of oral hygiene; Sig. = significance level; N = number of respondents. **p < 0.01 level.

## 4 Discussion

Maintaining satisfactory oral health for the mother during the pregnancy is crucial, and it plays a significant part in reducing the number of bacteria that are transmitted vertically from mothers to their children. In the current study, the correlation link between education levels and oral hygiene practices and knowledge was significantly revealed (*p*-value < 0.001), suggesting that an increase in knowledge of oral health may contribute to improved oral health practices. Singh and Prakash observed a similar result (26). Another study by Moawed et al. (27) demonstrated poor oral health knowledge, with a significant correlation between oral hygiene practices and educational levels. Recent studies in Saudi Arabia indicate that Saudi pregnant women demonstrate inadequate awareness of oral health, participate in substandard practices, and infrequently seek dental treatment (28). Likewise, another international study performed by Chacko et al. reported that the awareness of pregnant women and their practices regarding their own oral health and that of the newborn were inadequate and unaffected by their educational attainment (29). In contrast, Saddki et al. (30) revealed no significant correlation linked between education level and knowledge of oral hygiene among pregnant women. The results of the present study, based on overall statistical analysis, indicated that a majority of the Saudi women surveyed had an acceptable level of tolerance regarding the knowledge and practice of their oral hygiene. However, these results reveal a critical gap, because 30.4% of participants reported never brushing their teeth, and 42.6% believed that extra care is unnecessary during pregnancy. These numbers represent a major public health concern that cannot be ignored and demand attention. Many justifications exist for these observations in this study population, which most likely stem from a combination of limited access to prenatal dental counseling and deep-rooted cultural misconceptions toward oral health care during their pregnancy. Many of the women during pregnancy prioritized medical checkups while viewing dental care as optional during the pregnancy period. From the viewpoint of public health, these observations represent a lack of a critical component in current prenatal health programs. To bridge this gap, healthcare professionals, including gynecologists, nurses, and dentists, should collaborate to integrate basic oral health education directly into routine prenatal visits. Implementing educational programs during pregnancy may significantly reduce these gaps and prevent oral health issues in both mothers and their children. These observations highlight an emphasized need for different educational programs and prenatal counseling to improve maternal and child health outcomes.

Most Saudi women were acutely aware of the importance of visiting the dentist, oral care, and understanding the relationship between the mother’s oral health and that of her child. Additionally, most Saudi women understand which medications are not advisable during pregnancy and the importance of vitamins and supplements for the normal growth and development of a child. Furthermore, most dental procedures are considered safe during pregnancy periods, and the pregnant women should visit a healthcare provider (dentist) promptly in the event of a dental emergency, preferably in the middle interval of pregnancy (the second trimester) (17, 31). In the current study, 69.2% of the respondents understood that some medications are not recommended during pregnancy. This result aligns with the findings of a Saudi study by Almuhareb et al. (32), which showed positive knowledge, attitude, and practice toward using medications during pregnancy. Another study by Goruntla et al. (33) noted similar results. Moreover, 79.9% of respondents in the present study did not know that dental radiography and local anesthesia are safe during pregnancy. This result is consistent with findings by Bahanan et al. (34), which concluded a lack of understanding regarding the safety of dental radiographs during pregnancy. Another study by Sivasankari et al. (35) concluded that 40–80% of pregnant women exhibited insufficient understanding of the hazards and effects of dental radiation. Using protective precautions in dental clinics during X-ray imaging is critical for minimizing the risk of radiation exposure to the child (17, 36). The U. S. Food and Drug Administration classifies certain medications and anesthetics used in dental clinics as Category B, which has not been associated with fetal risks (17). Furthermore, 64.3% of the respondents demonstrated sufficient knowledge about the importance of using vitamins and supplements during pregnancy. This result is consistent with the findings of a previous study conducted by Xiang et al. (37). Utilization of nutritional supplements during pregnancy was high among Chinese women; they considered that supplementation could prevent or manage nutrition-related issues. Another study by Popa et al. (38) demonstrated that nutritional awareness exhibited a significant independent correlation with supplement utilization during pregnancy. However, several studies contradict the current findings (39, 40). A Saudi study by Ahmad et al. noted a lack of knowledge and attitude of pregnant women toward vitamin supplementation, including folic acid (39). Another study by Moran-Lev et al. (40) reported inadequacies in compliance with nutritional medical requirements during and post-pregnancy.

The current study demonstrated a significant positive correlation between oral hygiene practices and related knowledge. Improvements in oral health practices can be anticipated when a mother increases her oral hygiene knowledge. This result aligns with those of previous studies (28, 41, 42). Hebbal et al. (28) demonstrated a significant positive correlation between knowledge, practice, and the use of dental services. Moreover, Bokhari et al. (41) reported a beneficial association between oral health practices and knowledge. Jojo et al. (42) reported a limited positive correlation between knowledge and practice. Azizah et al. (43) drew a similar conclusion. However, many studies disagree with these findings (2, 44, 45). In a Saudi study, Gaffar et al. (2) reported pregnant women having essential oral health knowledge; however, this knowledge did not significantly correspond to their reported self-care practices. Bamanikar and Lee (44) reported substantial gaps in oral hygiene knowledge and practice among pregnant women. A Sudanese study by Ibrahim et al. (45) demonstrated the absence of an association between oral health knowledge and practice.

The present study has several methodological limitations. First, the utilization of self-reported data without clinical verification to verify oral hygiene status, such as plaque index or DMFT index measurements, could lead to inaccurate, uncorroborated responses by the participants. Second, the utilization of convenience and snowball sampling through social media platforms could introduce selection bias. Third, the small sample size includes a high proportion of participants with university education and unemployed participants; therefore, this sample cannot be considered representative of all pregnant women in Saudi Arabia, and the findings cannot be generalized to the entire population. Fourth, the study population was heterogeneous, as it included both currently pregnant women and those who had been pregnant before. The inclusion of previously pregnant participants may have introduced recall bias, which may have affected the validity and comparability of the data. Fifth, a number of important factors influencing oral health behaviors were not examined in this study, including household income, urban or rural residence, health insurance coverage, and access to dental services. Sixth, geographical information was lacking, as the exact regional distribution of the participants in Saudi Arabia was not documented in this study. These factors may influence knowledge and practices related to oral health, and the lack of detail about geography may hide regional differences. Our analysis was mainly focused on the educational level, and other sociodemographic variables were not exhaustively studied, thus limiting a more general understanding of the factors that influence oral health knowledge and practices. These factors should be assessed separately in future studies to appreciate their individual effects. Larger random sampling methods should be used, and the region of sample collection should be identified. Clinical examinations with the plaque index score and DMF index should be included as objective measures, and oral hygiene knowledge should be recorded through personal interviews to verify self-reported outcomes. Various demographic factors should be included in multivariable analyses to control for these variables. This study adds to the knowledge by identifying specific and critical gaps in maternal oral health care that are often masked by overall averages. The results indicate that future research should test the success of actual educational programs rather than awareness, and that practical strategies should include basic dental screening at initial prenatal visits and training prenatal nurses and doctors to provide basic oral health counseling.

## 5 Conclusion

Pregnant women in Saudi Arabia possess adequate knowledge concerning oral healthcare management. Most respondents demonstrated adequate oral health knowledge and favorable oral health practices. A strong positive correlation was observed between oral health knowledge and practice, indicating that pregnant women who were more aware of oral hygiene knowledge were more likely to report better oral hygiene practices. Furthermore, pregnant women demonstrated an adequate understanding of the potential impact of a mother’s overall health, including oral health, on the oral health of her child.

## Data Availability

The raw data supporting the conclusions of this article will be made available by the authors, without undue reservation.
